# Intraocular therapy in noninfectious uveitis

**DOI:** 10.1186/s12348-021-00267-x

**Published:** 2021-10-10

**Authors:** Rocco Luigi Modugno, Ilaria Testi, Carlos Pavesio

**Affiliations:** 1 Department of Neuroscience, Ophthalmology Unit, University of Padua, Padua, Italy; 2 Moorfields Eye Hospital, National Health Service Foundation Trust, 162 City Rd, Old Street, London, EC1V 2PD UK; 3 Biomedical Research Centre, Institute of Ophthalmology, UCL, London, UK

**Keywords:** Non-infectious uveitis, Local therapy, Intravitreal, Corticosteroids, Anti-vascular endothelial growth factor (VEGF), Immunosuppressive agents, Methotrexate, Biologics, Anti-tumor necrosis factor (TNF) α

## Abstract

Systemic corticosteroids and immunosuppressant agents are the mainstay of therapy for non-infectious uveitis (NIU). However, the risks associated with systemic administration and the need of delivering an effective and safe anti-inflammatory treatment targeted to the site of inflammation have prompt the use of local therapy in the management of NIU. This review will analyse the different local treatment options available, including corticosteroids, anti-vascular endothelial growth factor (VEGF), methotrexate and the recent biologics.

## Introduction

Non-infectious uveitis (NIU) encompasses a wide range of ocular inflammatory disorders, accounting for up to 20% of cases of severe vision impairment in adult population [1, 2]. There is great variability of clinical presentations, all associated with intraocular inflammation which results in cumulative damage to ocular tissues ultimately resulting in visual loss. Inflammatory cytokines, reactive oxygen species and lytic enzymes play a key role in structural and functional changes [3]. The main objective of our treatment strategies, which are currently non-specific, is to control active inflammation and try to prevent recurrences.

Systemic corticosteroids, eventually associated with second-line immunosuppressive agents, are the mainstay of treatment for non-infectious posterior uveitis (NIPU) [4, 5]. However, the well-known risks of side effects associated with systemic administration have prompted the adoption of local routes to directly deliver the drug to the site of inflammation [6]. Therapeutic strategies in NIPU vary based on the presence of systemic disease, type of ocular involvement, presence of systemic contraindications to medications, tolerance to therapy, patient’s needs and compliance. Local administration of corticosteroids is commonly indicated in patients with unilateral NIPU not associated with systemic disease, and is a reasonable alternative in case of resistance, intolerance or contraindications to systemic treatments. However, the use of local therapy in bilateral cases remains an option and the use of a combined approach may result in reduced burden of systemic therapies. Considering the ocular adverse events associated with the local use of corticosteroids, alternative strategies have been explored, including intravitreal anti-vascular endothelial growth factor (VEGF), methotrexate and more recently, biologics. This review will analyse the different local treatment options currently available for the management of NIPU.

## Corticosteroid*s*

Corticosteroids commonly administered via local route include triamcinolone acetonide (TA), dexamethasone (DEX) and fluocinolone acetonide (FA).

### Triamcinolone acetonide

TA has long been the most commonly used corticosteroid for treatment of unilateral, refractory macular oedema secondary to NIU [7–9]. It can be administered as suspension via intravitreal route or as periocular injection, either to the orbital floor or to the posterior sub-Tenon’ s space. The main advantage of intravitreal TA is the rapid delivery of a high concentration of a potent corticosteroid agent to the posterior segment of the eye, with subsequent immediate therapeutic effect. However, although effective in treating uveitis and macular oedema, its limited duration of effect (3 months) has resulted in a search for sustained-release intravitreal implants, which have since become more frequently used options [9].

### Sustained-release intravitreal implants

Compared to periocular and intravitreal TA, intravitreal corticosteroid implants offer the advantage of a gradual and sustained release of the drug to the posterior segment, resulting in reduced rates of relapses and re-injections.

#### Dexamethasone implant

DEX insert 0.7 mg (Ozurdex®, Allergan, Inc., Irvine, CA) is an intravitreal, biodegradable implant, approved for the treatment of macular oedema secondary to NIPU [10, 11]. The implant is injected through a preloaded applicator with a 22-gauge needle in an office-based setting. DEX has an approximately 12.5 time higher anti-inflammatory potency than TA and, because of its high solubility in water, it can achieve high concentration in the vitreous cavity (up to 3.0 mg/mL, compared to 1.2 mg/mL of TA) [12]. The implant gradually releases DEX for 6 months with the peak occurring 2 months after the injection.

Safety and efficacy of 0.7-mg and 0.35-mg DEX implants for treatment of intermediate and posterior NIU have been compared in a 26-week sham-controlled trial [13]. Eyes treated with DEX implants showed a significant improvement of vitreous haze (VH), best-corrected visual acuity (BCVA) and central retinal thickness (CRT) compared to the sham group. However, compared to the 0.35-mg group, the 0.7-mg DEX implant group had a significantly higher proportion of patients with absent VH at 8 weeks, coinciding with the peak of DEX concentration in the vitreous, whereas other outcome measures, including VA gain, CRT reduction and ocular adverse effects incidence, were not significantly different between the two dose groups [13].

The PeriOcular versus INTravitreal (POINT) trial compared the effectiveness of 40-mg periocular triamcinolone acetonide (PTA), 4-mg intravitreal triamcinolone acetonide (IVTA) and 0.7-mg intravitreal dexamethasone implant (IDI) for treatment of non-infectious uveitic macular oedema over a 6-month follow-up [14]. CRT and BCVA were significantly improved in all treatment groups at all follow-up visits, even though the extent of CRT reduction and BCVA gain were significantly higher in the intravitreal groups (IDI and IVTA) compared to the PTA group. Percentages of CRT reduction from baseline at week 8 were 23% in PTA group, 39% in IVTA group and 46% in IDI group. The intravitreal groups had a higher proportion of eyes with resolution of macular oedema. No significant difference regarding CRT and resolution of macular oedema was found between ITA and IDI group. Intravitreal treatment groups showed a greater increment in BCVA gain compared to the periocular one (4–7 letter at 4–8 weeks and 5 letters at 24 weeks, respectively). There was no significant difference in BCVA improvement between the intravitreal groups. Similarly, the proportion of eyes with intraocular pressure (IOP) ≥ 30 mmHg and eyes requiring IOP-lowering medications was similar in all treatment groups. However, the risk of having an IOP ≥ 24 mmHg or an IOP elevation ≥10 mmHg from baseline was higher for the intravitreal treatment groups compared to the PTA group (no significant difference was observed between the IDI and IVTA groups). There were no significant differences in the use of IOP medications between the three treatment groups at any time, even though the proportion of eyes treated with IOP medications increased throughout follow-up from 22% at randomization to 32% at 8-weeks and 39% at 24-weeks. No patient needed IOP-lowering surgery.

To summarize, the results suggest that intravitreal treatment is superior to periocular approach in the management of macular oedema and improvement of visual acuity, and that the efficacy between intravitreal treatments, namely TA and DEX implant, is comparable [14]. In this regard, intravitreal therapy is increasingly preferred to periocular steroids which, however, remain a viable therapeutic option for patients who are aphakic and for whom intravitreal injections are not an option.

Although macular oedema is the most frequent indication to intravitreal corticosteroid implants in the management of NIPU, it is not the only one. A multicenter retrospective cohort study on intravitreal corticosteroid implants in NIPU showed that vitritis is the second most common indication, confirming the use of local corticosteroids devices with the aim of achieving control of both macular oedema and vitreous inflammation [15]. Similarly, the effectiveness of intravitreal corticosteroids implants in controlling intraocular inflammation has been repeatedly reported in cases of retinal vasculitis [16–19].

#### Fluocinolone acetonide implants

FA implants are available at different doses: 0.59 mg (Retisert®, Bausch and Lomb, Inc.), 0.18 mg (Yutiq®, Eyepoint Pharmaceuticals, Inc.) and 0.19 mg (Iluvien®, Alimera Sciences, Inc.). Despite the short systemic half-life of FA, the non-biodegradable FA implant (FAi) releases the steroid at a stable rate for up to 3 years, a much longer period of action than DEX and TA [12, 20].

##### 0.59 mg fluocinolone acetonide implants

Intravitreal 0.59-mg FA implant (Retisert®) was the first FDA-approved implant for treatment of NIPU. The implant was intravitreally placed through a pars plana sclerotomy and then fixed to the sclera. It releases FA over a period of 3 years at a rate of 0.6 μg/day during the first month and 0.3–0.4 μg/day for about 30 months.

The Multicenter Uveitis Steroid Treatment (MUST) trial compared efficacy and safety of systemic corticosteroids (plus immunosuppressants when indicated) and 0.59-mg FAi in 479 eyes with NIPU over a 24-month follow-up period [21]. The results showed that both approaches are effective with no evidence of a clear superiority of either of them [21]. Both the implant and systemic groups showed improvement in intraocular inflammation, BCVA and control of macular oedema at 24 months, with no statistically significant difference between the groups. However, control of intraocular inflammation at 24 months was more frequent in the implant-treated group than in systemic corticosteroids-treated patients (88% vs. 71%, respectively). In addition, there was a significantly greater improvement of self-reported quality of life in the FAi than in the systemic corticosteroids group. However, a significantly higher proportion of FAi-treated eyes showed cataract development and/or IOP elevation.

The MUST Follow-up Study followed 248 eyes enrolled in the MUST Trial for up to 7 years with a focus on long-term outcome of macular oedema [22]. Overall, within the 7-year follow-up, 94% of eyes resolved macular oedema with a median time to resolution of 1.09 years, while cumulative proportion with relapsed macular oedema was 43%. Compared to the systemic corticosteroid group, the hazard for relapse of macular oedema was lower in the FAi group within 3 years of implantation, even though it did not reach a statistical significance. On the contrary, it was significantly higher in the FAi than systemic group 3 years after implantation. However, eyes in the systemic treatment group with resolved macular oedema required adjunctive short-acting regional corticosteroid injections at a significantly higher rate than the FAi treatment group during long-term follow-up [22].

Similarly, a randomized clinical trial compared the effectiveness of 0.59-mg FAi and systemic therapy (corticosteroids ± immunosuppressive agents) in 140 patients with NIPU analysing time of first uveitis recurrence at 24 months [20]. Subjects in the FAi group showed a significantly lower recurrence rate and mean number of post-treatment recurrences compared to the systemic group (18.2% vs. 63.5% and 0.3 vs. 1.2, respectively). The rate of VH and macular oedema reduction at 24 months was statistically higher in the FAi group but the mean BCVA at 24 months was similar between the two study groups. The results thus, suggest that, despite the two approaches have comparable functional outcomes, 0.59-mg FAi is more effective in controlling inflammation in terms of reduction of recurrence rate and number of recurrences compared to systemic treatment [20]. However, ocular side effects, including cataract requiring surgical extraction (87.8% FAi group vs. 19.8% systemic group) and IOP elevation requiring medical (62.1% vs. 20.3%) or surgical (21.2% vs. 2.7%) treatment, were significantly more frequent in the FAi than in the systemic one.

Since 0.59-mg FA implant requires surgical implantation with associated risks of post-surgical hypotony, resistant IOP elevation requiring device explant, scleral thinning, endolphthalmitis and separation of the implant from the strut, new corticosteroids intravitreal implants have been introduced, utilising a simpler delivery method and overcoming some of these limitations.

##### 0.18 and 0.19 mg fluocinolone acetonide implants

Both 0.19-mg (Iluvien®) and 0.18-mg (Yutiq®) FA implants are intravitreally injected through the pars plana with a preloaded applicator with a 25-gauge needle in an office-based setting. Both are designed to release FA over a 3-year period at a rate of 0.2 μg/day, progressively decreasing to 0.1 μg/day.

The three-year results of the prospective, randomized, sham injection-controlled clinical trial analysing the recurrence rate of uveitis in adults with a diagnosis of NIPU treated with intravitreal 0.18-mg Fai compared to the standard of care have been recently published [23, 24]. The recurrence rate of uveitis was statistically lower in the implant group compared to the sham group at 6 months (27.6% vs. 90.5% respectively), 12 months (37.9% vs. 97.6%) and 36 months (65.5% vs. 97.6%), as well as was the mean number of recurrences per eye (0.7 vs. 2.5 at 12 months and 1.7 vs. 5.3 at 36 months, respectively). A significant higher proportion of eyes in the FAi group had no recurrences compared to the sham group (34.5% vs. 2.4%, respectively), or had only one recurrence in 36 months (33.3% vs. 11.9%, respectively). Median time to first recurrence was significantly longer in the implant group than in the sham group (378 vs. 70.5 days at 12 months and 657.0 vs. 70.5 days at 36 months, respectively). A decrease in BVCA of ≥15 letters from baseline was less common in the FAi group compared to the sham group (14% vs. 31% at 12 months and 1.4% vs. 8.8% at 36 months, respectively), as well as was an increase in BVCA of ≥15 letter from baseline (33.3% vs. 14.7% at 36 months, respectively). Mean central subfield thickness (CST) decreased more in the FAi group than in the sham-injected eyes at day 28 (− 61.3 vs. -7.5 μm, respectively), and a higher proportion of FAi-treated patients had no macular oedema at 12 months compared to patients treated with sham injections (71% vs. 48%, respectively). Although not statistically significant, fewer eyes in the FAi-treated group had persistent macular oedema at 36 months compared to sham-treated eyes (13.0% vs. 27.3%, respectively). A similar percentage of eyes in the FAi- and sham-treated groups had no VH or anterior chamber cells at month 36; however, the control of intraocular inflammation was achieved more rapidly in the FAi-treated eyes. There was a statistically significant difference in the proportion of patients receiving adjunctive systemic corticosteroids or immunosuppressants among the two study groups (19% FAi group vs. 40% sham group at 12 months and 57.5% vs 97.6% at 36 moths, respectively). Regarding the safety, mean change from baseline IOP was slightly greater at 12 months in the FAi group (+ 1.3 mmHg) than in the sham injection (+ 0.2 mmHg) eyes. FAi treated patients had a higher rate of IOP ≥ 25 mmHg compared to the sham group (17% vs. 5%, respectively), or of IOP increase > 5 mmHg over baseline (42% vs. 14%, respectively). At 36 months, a higher proportion of FAi-treated eyes received IOP-lowering medication compared to the sham-treated eyes (42.5% vs. 33.3%, respectively), while surprisingly, the proportion of eyes that underwent IOP-lowering surgery was lower in the FAi-treated group then in the sham group (5.7% vs. 11.9%, respectively). This was explained by the use of rescue therapy with other forms of local steroids predominantly in the sham arm of the study. Cataract surgery was more frequently required in the FAi-treated group compared to the sham-treated group (73.8% vs. 23.8% of eyes, respectively) [23, 24]. To summarize, the study demonstrated that 0.2 μg/day intravitreal sustained-release of FA is a safe and effective treatment for chronic and recurrent NIPU, being associated with a substantial lower recurrence rate and number of recurrences, and longer recurrence-free time compared to the standard of care. This results in less retinal structural and functional damages, less frequent examinations, higher patient’s adherence to therapy and better quality of life [25]. Furthermore, the use of a lower dose of FA compared to the initial dose of 0.59-mg allows a reduction in the risk of IOP elevation.

Given the lack of comparative trials between low-dose FA and DEX implants and long-term studies on repeated injections of DEX implants, it is difficult to draw conclusions about which implant is better in terms of efficacy and safety in the treatment of NIU [5, 24]. It is important to stress that these implants have been licensed with different indications, especially, the FA implant is indicated with the objective of preventing relapses and not to treat active inflammation, different from the DEX implant. The longer anti-inflammatory effect of low-dose FAi compared to DEX implant makes FAi more effective in the prevention of relapses.

## Intravitreal anti-vascular endothelial growth factors (VEGF)

Intravitreal anti-VEGF agents are diffusely used to treat choroidal/retinal neovascularization and cystoid macular oedema (CMO) secondary to degenerative, vascular and inflammatory diseases. VEGF takes part in the inflammatory response by increasing vascular permeability and contributing to the blood-retinal barrier disruption that lead to CMO, the most common cause of visual impairment in uveitis [26]. Mean levels of VEGF are significantly higher in aqueous humor of patients with uveitic macular oedema than in patients with uveitis and no intraretinal fluid [27]. This explains the rationale for the use of anti-VEGF in patients with recalcitrant uveitic macular oedema and in those with contraindications for the use of local corticosteroids (e.g. steroid-responders). Anti-VEGF has limited anti-inflammatory action, and thus, in the presence of macular oedema and active intraocular inflammation, it is important to use therapy to control inflammation at the same time.

A number of studies on anti-VEGF use in the treatment of uveitic macular oedema has been published, although the retrospective design, small sample size, different protocols used and lack of direct comparison among anti-VGEF agents make it difficult to interpret often conflicting results. In a retrospective case series of 13 patients with recalcitrant uveitic macular oedema, a single intravitreal injection of 2.5-mg bevacizumab (Avastin®, Genentech, Inc., South San Francisco, CA) resulted in a significant reduction of CRT. However, there was no improvement of BCVA at 3 months [28]. By contrast, both CRT and BCVA were significantly improved 4 weeks after a single injection of 1.25-mg bevacizumab in a case series of 11 patients with uveitic macular oedema. These conflicting results might be explained by the grade of macular and/or optic disk leakage on fluorescein angiography, reflecting the grade of blood-retinal barrier disruption. Eyes with extensive leakage derived from a higher grade of blood-retinal barrier disruption can potentially be less likely to respond to anti-VEGF therapy [29]. In a retrospective analysis of patients treated with a median of two injections of 1.25-mg bevacizumab, Lott at al. found that neither BCVA nor CRT significantly changed during the follow-up [30]. Conversely, Meckensen et al. found a significant reduction of CRT at 6–8 weeks in patients with persistent uveitic macular oedema treated with 1.25-mg or 2.5-mg bevacizumab. However, to maintain the effect, patients in 1.25-mg group required more frequently a second injection at 4 weeks compared to the patients in 2.5-mg group. These results suggest a comparable efficacy and safety for both dose groups even though patients injected with 2.5-mg bevacizumab showed an apparently longer effect [31].

Other studies compared intravitreal anti-VEGF agents with intravitreal TA. A single injection of 4-mg intravitreal TA (IVTA) and 2.5-mg bevacizumab were retrospectively compared in recalcitrant uveitic macular oedema over a 6-month follow-up period. CRT significantly improved in both groups, while a significant BVCA improvement was observed only in IVTA-treated patients [32]. Opposite results were described in a prospective clinical trial including two study groups receiving 1 to 3 intravitreal injections of 1.25-mg bevacizumab and 1 to 3 intravitreal injections of 2-mg TA respectively during a 36-month follow-up period. BCVA significantly improved in both groups, while CRT and fluorescein leakage reached a statistical reduction only in IVTA-treated patients [33]. In a similar and retrospective study, Bae et al. compared the effect of 1.25-mg intravitreal bevacizumab, 4-mg intravitreal TA and 40-mg posterior sub-Tenon TA. CRT and BCVA significantly improved from baseline at 4 weeks in all groups, while any significant difference was observed among the groups themselves. The effect progressively declined with time at different rates: the median period of effect was 12 weeks for posterior sub-Tenon TA, 16 weeks for intravitreal bevacizumab and 30 weeks for intravitreal TA [34]. Intravitreal ranibizumab (Lucentis®, Genentech, Inc., South San Francisco, CA) has been also successfully used for the treatment of recalcitrant uveitic macular oedema [35, 36].

Altogether the result support the use of intravitreal anti-VEGF agents as alternative or supplementary treatment for recalcitrant uveitic macular oedema. Compared to intravitreal corticosteroids, anti-VEGF agents are associated with a significantly lower risk of cataract development and IOP elevation, although characterized by limited anti-inflammatory effect and short intravitreal half-life requiring monthly injections [29, 32].

## Intravitreal immunomodulating agents

### Methotrexate

Methotrexate (MTX) is a steroid-sparing agent, commonly administered via systemic route in inflammatory diseases with or without ocular involvement. MTX is an antimetabolite that interferes with the metabolism of folic acid and thus with DNA synthesis, inhibiting the dihydrofolate reductase. Intravitreal MTX was first used in the treatment of primary intraocular lymphoma. Given its anti-inflammatory effect, the drug has been used and proven effective as an alternative option in patients with NIPU and/or macular oedema difficult to treat with conventional therapy [37, 38]. In addition, thanks to its anti-angiogenic effect, MTX has been also used to treat choroidal neovascularization refractory to repeated injections of anti-VEGF agent [39, 40].

Efficacy of a single intravitreal injection of 400 μg-MTX in 0.1 ml has been evaluated in a case series of 15 patients with unilateral exacerbations of NIPU and /or macular oedema over a 6-month follow-up period [41]. MTX injections were followed by a significant improvement of BCVA, CRT and VH starting by week 1 with a median time to relapse of 4 months. In addition, about a half of patients on concomitant systemic corticosteroids were able to substantially reduce the dose after MTX intravitreal injection [41]. Although intravitreal 400 μg-MTX appears as a safe and effective alternative to intravitreal corticosteroids, randomized clinical trials with larger samples and longer follow-up are needed to establish its therapeutic role in the management of NIU. Compared to intravitreal corticosteroids, intravitreal MTX is less likely associated with IOP elevation and cataract development. However, corneal epitheliopathy is a known reported side effect [41]. The short duration of action, which implies the need for repeated injections, is one of the main disadvantage compared to corticosteroids sustained-release implants. To date, sustained-release MTX implants have been only tested in rabbits [42].

### Sirolimus

Sirolimus, also known as rapamycin, is a macrolide with antifungal, anti-inflammatory and immunosuppressive effects on T-lymphocytes [43]. Oral sirolimus is commonly used to prevent kidney transplant rejection, but intravitreal administration of the drug has been considered for treatment of refractory NIPU. The Sirolimus as a therapeutic Approach uVEitis (SAVE) study prospectively evaluated patients with non-infectious intermediate, posterior or panuveitis receiving three injections of 352-μg intravitreal or 1320-μg subconjunctival sirolimus at 2-month intervals during a 12-month follow-up period. A significant reduction of VH and dosage of concomitant systemic corticosteroids was seen in both groups at 12 months, while changes of BCVA and CRT from baseline were not statistically significant [44] In the SAVE-2 study, eyes receiving 6 injections of 440-μg sirolimus at monthly intervals was compared with eyes receiving 3 injections of 880-μg sirolimus at bi-monthly intervals [45]. VH was significantly reduced at 6 months in both groups, but no significant difference was observed between the low- and high-dose group. Conversely, CRT and BCVA didn’t significantly changed from baseline in both the groups. Altogether these results suggest that subconjunctival and intravitreal injections are both safe and effective in reducing ocular inflammation and that the injection of 880-μg sirolimus every 8 weeks is not superior to the injection of 440-μg dose every 4 weeks in terms of inflammation control and duration of the anti-inflammatory effect [44, 45].

The Sirolimus Study Assessing Double-masKed Uveitis TReAtment (SAKURA) included a total of 592 patients (347 patients in SAKURA 1 and 245 patients in SAKURA 2) randomly assigned to receive three injections of 44 μg (low-dose active control), 440 μg or 880 μg intravitreal sirolimus at by-monthly intervals [46–48]. In both SAKURA 1 and 2, the proportion of patients with no VH at month 5 was significantly higher in the 440-μg group than in the 44-μg group, but not in the 880-μg group compared to the 44-μg group. BCVA was preserved in a comparable proportion of patients in all dose groups (79% in 44-μg group; 80.4% in 440-μg group; 80% in 880-μg group) with a greater improvement from baseline in patients with the worst baseline BCVA. The majority of patients had an improvement in CRT ≥ 50 μm at 5 months, with a higher proportion in the 440-μg dose group and among subjects without epiretinal membrane or posterior hyaloid membrane traction. Patients in the 440-μg dose group also showed the highest proportion of corticosteroids tapering success and lowest proportion of rescue therapy before month 5. The incidence adverse events was similar across the dose groups with iridocyclitis being the most common ocular side effect [46, 47].

Despite the limited data available, patients receiving intravitreal sirolimus are unlikely to develop cataract or glaucoma, even though comparative studies between sirolimus and corticosteroids have been not performed. Although characterized by anti-inflammatory activity which makes the sirolimus suitable as alternative treatment for recalcitrant NIU with or without macular oedema, its short half-life requiring multiple re-injections is one of its main disadvantages. To date, the safety of a biodegradable intravitreal sirolimus implant has been tested in rabbits with promising results [49].

## Intravitreal anti-TNFα agents

Monoclonal antibodies against TNFα, a key pro-inflammatory cytokine, are diffusely used for the treatment of eye-involving systemic inflammatory diseases [50, 51]. Until now, the intravitreal administration of anti- TNFα agents has been only evaluated by a limited number of studies, including few patients with no standardized protocol. In the first pivot study, seven patients with refractory NIU were treated with a single intravitreal injection of 1.5 mg / 0.15 mL infliximab, a chimeric anti-TNFα antibody [52]. BCVA, CRT and VH were all significantly improved at 4 weeks after injection and none of the patients reported ocular or systemic side effects. Previous studies had already established the safety of 1 mg to 2 mg intravitreal infliximab on animal models [53, 54]. Another study evaluated efficacy and safety of a single intravitreal injection of 1 mg / 0.05 mL infliximab over a 4-week follow-up period in patients with sight-threatening relapsing uveitis in Behçet disease. BCVA, CRT, VH, anterior chamber cells, retinitis and vasculitis were significantly improved by day 7 and continued to improve through day 30, while none of injected patients reported ocular or systemic adverse events [55]. Even less is known about intravitreal adalimumab (Humira, Abbott Laboratories, Abbott Park, IL), a fully human anti-TNFα antibody. Intravitreal injection of 0.5 mg / 0.05 mL adalimumab at monthly intervals for 3 months in eight patients with chronic and refractory uveitic macular oedema didn’t show any significant change of BCVA and CRT as well as any ocular and systemic adverse events [56]. Conversely, six patients with active NIU treated with 2 injections of 1.5 mg / 0.05 mL adalimumab at 2-week intervals followed by 6 monthly injections showed a significant improvement of BCVA, CRT and fluorescein angiography score at 26 weeks [57]. The studies are difficult to compare and provide contrasting results on intravitreal anti-TNFα agents as therapeutic alternative for refractory NIPU.

## Conclusion

Intravitreal treatments, in particular corticosteroids, have gained a prominent role in the treatment of NIU. The use of slow-release devices has overcome the limitations of repeated injections and still represent an important option in the management of NIPU. Current available options for intraocular therapy result in control of inflammation for periods ranging from 3 to 36 months. Even though the price of the devices is high when considering individual use, the long effect and the benefit of avoiding systemic therapy with all possible side effects and the need for regularly monitoring of the patients, which includes several hospital visits, blood tests and use of preventative therapy for known side-effects such as osteoporosis, makes this option an attractive alternative in terms of health economics. Cataract surgery is a common complication when using local steroids, but it also represents a common problem in uveitis patients in general.

The use of local therapy has been predominantly used for patients with unilateral or asymmetric disease and for those without an active systemic disease. These are obvious indications, as long as there are no contraindications such as steroid induced ocular hypertension/glaucoma. Bilateral cases may also benefit from this strategy both as a primary therapy in cases of contraindication or intolerance to systemic therapy and as an adjunct to help reduce the burden of systemic therapy. This is something that became quite obvious in the management of Birdshot retinochoroiditis where the use of a dual approach has allowed good control of the retinal vasculitis and choroiditis with the use of reduced systemic therapy (unpublished data). Ozurdex is the choice for local therapy in cases of active disease, including macular oedema, and Iluvien becomes the option for those who need long-term control using local approach. A good response to Ozurdex is reassuring but does not guarantee an equal response to Iluvien.

Despite their anti-inflammatory efficacy, the well-known ocular side effects, the occurrence of recalcitrant inflammation and the presence of contraindications to corticosteroids have prompted the search for alternative, nonsteroidal therapies. An increasing number of intravitreal drugs is being studied for NIU treatment, including anti-VEGF, immunomodulating and biological agents, with encouraging results. Randomized clinical trials are needed to reliably establish their efficacy, safety and indications.

## Data Availability

Not applicable.

## Abbreviations

NIU: Noninfectious uveitis
VEGF: Vascular endothelial growth factor
TNF: Tumor necrosis factor
NIPU: Noninfectious posterior uveitis
TA: Triamcinolone acetonide
DEX: Dexamethasone
FA: Fluocinolone acetonide
VH: Vitreous haze
BCVA: Best-corrected visual acuity
CRT: Central retinal thickness
PTA: Periocular triamcinolone acetonide
IVTA: Intravitreal triamcinolone acetonide
IDI: Intravitreal dexamethasone implant
IOP: Intraocular pressure
FAi: Fluocinolone acetonide implant
CST: Central subfield thickness
CMO: Cystoid macular oedema
MTX: Methotrexate
